# Mitochondrial Homeostasis Regulating Mitochondrial Number and Morphology Is a Distinguishing Feature of Skeletal Muscle Fiber Types in Marine Teleosts

**DOI:** 10.3390/ijms25031512

**Published:** 2024-01-26

**Authors:** Busu Li, Huan Wang, Xianghui Zeng, Shufang Liu, Zhimeng Zhuang

**Affiliations:** 1 National Key Laboratory of Mariculture Biobreeding and Sustainable Goods, Yellow Sea Fisheries Research Institute, Chinese Academy of Fishery Sciences, Qingdao 266071, China; libusu1616@163.com (B.L.); wanghuan@ysfri.ac.cn (H.W.); zengxianghui@wie-biotech.com (X.Z.); zhuangzm@ysfri.ac.cn (Z.Z.); 2 Laboratory for Marine Fisheries Science and Food Production Processes, Laoshan Laboratory, Qingdao 266237, China

**Keywords:** *Takifugu rubripes*, slow-twitch muscles, fast-twitch muscles, mitochondrial homeostasis, mitochondrial adaptation

## Abstract

Fishes’ skeletal muscles are crucial for swimming and are differentiated into slow-twitch muscles (SM) and fast-twitch muscles (FM) based on physiological and metabolic properties. Consequently, mitochondrial characteristics (number and morphology) adapt to each fiber type’s specific functional needs. However, the mechanisms governing mitochondrial adaptation to the specific bioenergetic requirements of each fiber type in teleosts remain unclear. To address this knowledge gap, we investigated the mitochondrial differences and mitochondrial homeostasis status (including biogenesis, autophagy, fission, and fusion) between SM and FM in teleosts using *Takifugu rubripes* as a representative model. Our findings reveal that SM mitochondria are more numerous and larger compared to FM. To adapt to the increased mitochondrial number and size, SM exhibit elevated mitochondrial biogenesis and dynamics (fission/fusion), yet show no differences in mitochondrial autophagy. Our study provides insights into the adaptive mechanisms shaping mitochondrial characteristics in teleost muscles. The abundance and elongation of mitochondria in SM are maintained through elevated mitochondrial biogenesis, fusion, and fission, suggesting an adaptive response to fulfill the bioenergetic demands of SM that rely extensively on OXPHOS in teleosts. Our findings enhance our understanding of mitochondrial adaptations in diverse muscle types among teleosts and shed light on the evolutionary strategies of bioenergetics in fishes.

## 1. Introduction

Skeletal muscle is a type of tissue that can contract and produce force, enabling movement. It requires a steady supply of energy in the form of ATP, which is stored intramuscularly and generated via either anaerobic or aerobic metabolism [1]. Skeletal muscle can be classified as either slow- or fast-twitch fiber. Slow-twitch fibers are used for posture control and prolonged, submaximal (aerobic) exercise, leading to a prevalence of aerobic metabolism. Fast-twitch fibers, on the other hand, produce greater tension and force for shorter durations but tire out more quickly, relying predominantly on anaerobic metabolism. Due to the contrasting energy metabolic processes and physiological characteristics of slow- and fast-twitch fibers, there are significant variations in the mitochondrial content and morphology between these two muscle fibers across different species [2]. Mitochondria, the cell’s energy regulators, can change in number and structure to adapt to the energy needs of different muscles [3]. In mammals, slow-twitch (oxidative) muscles have a lot of long, connected mitochondria spread across many sarcomeres. In contrast, fast-twitch muscles (glycolytic) have fewer, separate mitochondria arranged in rows and columns. This adjustment shows the unique characteristics of mitochondria in different muscle types [4]. 

Mitochondrial content and morphology maintenance in skeletal muscles is reported to depend on the mitochondrial homeostasis containing biogenesis, autophagy, and dynamics (fusion/fission) in mammals. The continuous alternation of mitochondrial homeostasis facilitates the adaptation of the mitochondrial network to the distinct functional needs of different muscle types, achieved by regulating mitochondrial number, morphology, and distribution [5]. 

Mitochondrial biogenesis is the process of generating new mitochondrial material from pre-existing mitochondria, resulting in the increase of both mitochondrial number and size [6,7]. Mitochondrial autophagy, a process that specifically recycles damaged mitochondria, helps maintain a healthy state of mitochondrial turnover [8]. Mitochondrial fusion triggers the generation of interconnected, extensive mitochondrial networks and increases the mitochondrial area. This process enables the mixing and redistribution of mitochondrial contents including mtDNA and proteins [9]. Conversely, mitochondrial fission contributes to the proper distribution of mitochondria in response to ATP demand by creating new mitochondria, and also facilitates the segregation of damaged mitochondria. Mitochondrial fission can maintain mitochondrial heath and increase the number of mitochondria [10]. In mice, it was observed that slow-oxidative muscles displayed higher rates of mitochondrial biogenesis and autophagy than fast-glycolytic muscles by detecting molecular markers of mitochondrial turnover [11]. Also, mitochondrial fusion and fission was reported as a distinguishing feature of different skeletal muscle fibers that is tailored to the fibers’ physiology. Mitochondrial fusion is the coordination of the outer mitochondrial membrane (OMM) and inner mitochondrial membrane (IMM)’s fusion, mediated by mitofusins (MFN) and optic atrophy protein 1 (OPA1), respectively. Mitochondrial fission is triggered by the dephosphorylation and subsequent recruitment of DRP1 (dynamin-related protein 1) and dynamin 2 (DNM2) to the mitochondrial surface, where they bind to their adaptor, mitochondrial fission factor (MFF) [12,13]. Slow-twitch muscle performed fusion at a higher rate compared to fast-twitch muscle [4]. Actually, slow- and fast-twitch muscle displays a mosaic distribution within the skeletal muscles of mammals, which makes mammals a poor study model for these two muscle types [14]. Previous studies of skeletal muscles in mammals often used anatomically distinct muscles as representative examples of these two fiber types. For instance, the soleus muscle and diaphragm muscle are commonly considered as oxidative slow-twitch muscles, while the extensor digitorum longus (EDL) muscle and tibialis anterior muscle (TA) are typically considered as fast-twitch muscles [4,11]. 

Unlike mammals, teleost fish have anatomically distinct slow-twitch muscles (SM) and fast-twitch muscles (FM) that are easily distinguishable by color. Hence, when compared to mammals, teleost fish are an ideal model for studies on mitochondrial differences between SM and FM. Although studies have shown that certain fish species, such as Tilapia [15], exhibit higher mitochondrial numbers in SM than FM, the regulatory mechanisms governing these mitochondrial phenotypes remain inadequately understood. In this study, utilizing *Takifugu rubripes* as the research subject, our objective is to elucidate the mechanisms underlying mitochondrial homeostasis and its regulation of mitochondrial number and morphology. This investigation aims to provide insights into how these adaptations effectively meet the cellular bioenergetic requirements of both slow- and fast-twitch muscles. *T. rubripes* is a commonly found fish in the Yellow Sea, with a fully sequenced genome [16] and extensive research—including transcriptome analysis—on its SM and FM [17,18,19,20]. In addition, its anatomically distinct distribution of different types of skeletal muscles makes *T. rubripes* an exceptional model for investigating mitochondrial networks in different skeletal muscle types. This model offers a valuable opportunity to advance our comprehension of the mitochondrial homeostasis process and its pivotal role in regulating mitochondrial number and morphology in teleosts. This, in turn, facilitates the adaptation of mitochondria in both slow-twitch (SM) and fast-twitch (FM) muscles among teleosts with diverse swimming performances. Therefore, we firstly analyzed the number and morphology of mitochondria in these two muscle types. Since oxidative phosphorylation occurs in the mitochondria, we subsequently compared the expression levels of genes related to aerobic and anaerobic metabolite processes, which are associated with the mitochondrial numbers of these two muscle types. Finally, we determined the mitochondrial homeostasis status, including biogenesis, fusion, fission, and autophagy, between slow- and fast-twitch muscles. This allowed us to obtain a more profound comprehension of the mitochondrial homeostasis mechanisms responsible for maintaining mitochondrial number and morphology, and how they contribute to the adaptation of different muscle types to their respective energy requirements in teleosts.

## 2. Results

### 2.1. Histological Characteristics of SM and FM of T. rubripes

**Figure 1:**
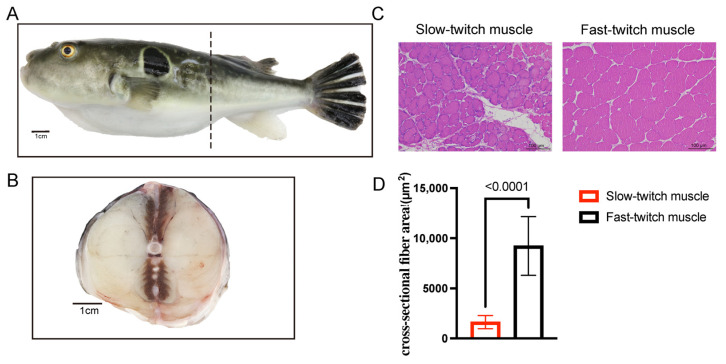
Slow-twitch muscles and fast-twitch muscles distribution and histochemical demonstration of adult *T. rubripes*, *n* = 3. (**A**) The dotted line shows a cross-section near the cloaca of *T. rubripes*. (**B**) Cross section of *T. rubripes*. (**C**,**D**) Representative images of hematoxylin–eosin staining and fiber area calculation of slow- and fast-twitch muscle of *T. rubripes*.

Within a cross-sectional view of *T. rubripes*, the red SM and white FM were easily identified based on their distinct muscle colors. The higher concentration of the pigment myoglobin imparts a red color to slow-twitch muscles. As shown in Figure 1A,B, fast-twitch muscles comprise the majority of trunk muscle, and slow-twitch muscles are located at the dorsoventral midline and adjacent to the spine in *T. rubripes*. According to the histology and image analysis, we found that the area of slow-twitch muscle fibers was 1634 ± 654.743 μm^2^, while the area of fast-twitch muscle fibers was 9233 ± 2920.65 μm^2^ (Figure 1C,D). The area of slow-twitch muscle fibers was smaller than that of fast-twitch muscle fibers in *T. rubripes*, which was consistent with other teleosts such as *Paralichthys olivaceus*, *Seriola dumerilii*, and *Liza aurata* [21,22]. 

### 2.2. SM Contain More Mitochondria Than FM in T. rubripes

**Figure 2:**
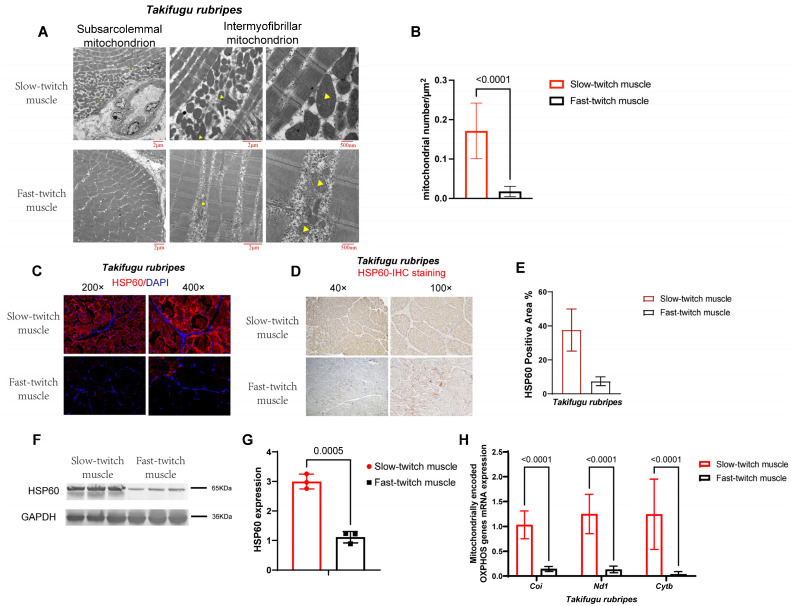
Slow-twitch muscles contain more mitochondria than fast-twitch muscles in *T. rubripes*, *n* = 3. (**A**) Representative transmission electron microscopic ultrastructural images and mitochondrial number calculation (**B**) of slow- and fast-twitch muscles. The yellow arrowheads indicate mitochondria. (**C**–**E**) Mitochondrial-specific protein (HSP60) staining and positive area calculation of slow- and fast-twitch muscles. Data in panel (**E**) is calculated from panel (**D**). (**F**,**G**) Immunoblot (**F**) and densitometric quantification (**G**) of HSP60 protein. (**H**) Mitochondrial-encoded OXPHOS gene expression detected through q-RT-PCR. *p* values are indicated using Student’s *t*-test.

We observed the ultrastructure of mitochondria through TEM analysis. As shown in Figure 2A, mitochondria are located beneath the sarcolemma and between myofibrils in skeletal muscles, and the mitochondrial number of SM was relatively higher than FM (Figure 2B). Immunohistochemical staining revealed that the positive area of mitochondrial specific protein HSP60 in slow-twitch muscles was greater than that in fast-twitch muscles (Figure 2C–E). This was further supported by HSP60 western blotting, which indicated that its expression level in SM was also significantly higher than FM (Figure 2F,G). Next, we assessed the mRNA expression level of mitochondrial genome encoded genes, including OXPHOS complex III subunit cytochrome b (*Cytb*), complex IV subunit cytochrome c oxidase 1 (*COI*), and complex I subunit NADH dehydrogenase 1 (*ND1*). As shown in Figure 2H, all of these mitochondrial genome-encoded OXPHOS genes were highly expressed in slow-twitch muscles. Therefore, the above results summarized that slow-twitch muscles contained larger mitochondrial numbers than fast-twitch muscles, which was certified by TEM images, mitochondrial specific protein expression, and mitochondrial genome-encoded gene expression.

### 2.3. Gene Signatures of Energy Metabolism Are Different between SM and FM 

The immediate energy source during muscle contraction is ATP, which is generated through anaerobic and aerobic metabolism. Anaerobic pathways react rapidly and provide the necessary support for explosive contractions, whereas aerobic pathways moderately increase energy provision, predominating during sustained submaximal exercise [23,24]. Mitochondria are the primary site of aerobic metabolism, while anaerobic metabolism occurs in the cytoplasm. The expression levels of genes related to energy metabolism can provide insight into differences in mitochondrial numbers between SM and FM. Here, we compared the expression levels of genes involved in aerobic and anaerobic metabolism in SM and FM using transcriptome data of *T. rubripes* [19].

**Figure 3:**
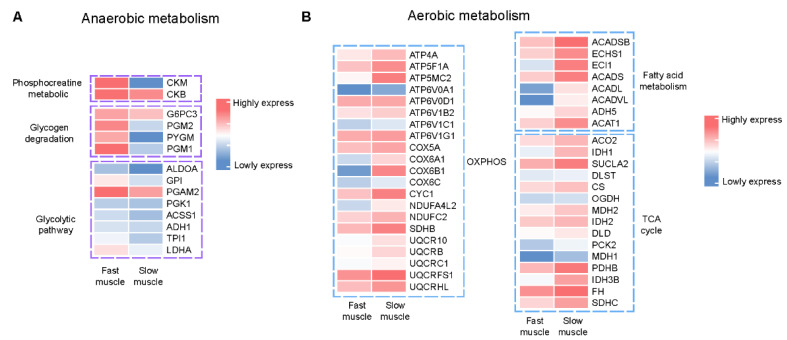
Heatmap of signature genes of anaerobic (**A**) and aerobic (**B**) metabolism between slow- and fast-twitch muscles of *T. rubripes*.

The predominant anaerobic pathways to generate ATP encompass degradation of phosphocreatine (PCr), the breakdown of muscle glycogen, and glycolysis. On the other hand, fatty acid metabolism, the TCA cycle, and oxidative phosphorylation are the dominating ATP-producing systems of aerobic metabolism. The transcriptome data showed that gene sets involved in anaerobic metabolism were highly expressed in FM (Figure 3A), while genes involved in aerobic metabolism were highly expressed in SM (Figure 3B). These results indicate that slow-twitch muscles, which contain more mitochondria, perform more aerobic metabolism compared with fast-twitch muscles, which depend more on anaerobic metabolism. 

### 2.4. Slow-Twitch Muscle Performs Higher Mitochondrial Biogenesis in T. rubripes Than Fast-Twitch Muscle

**Figure 4:**
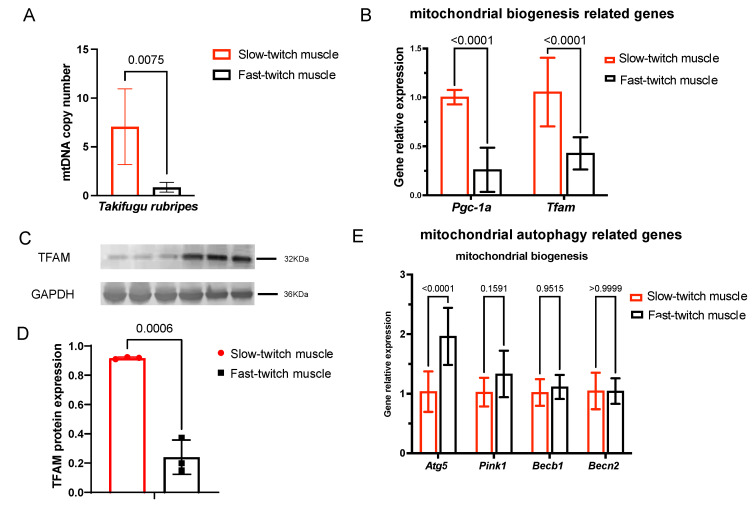
Slow-twitch muscles exhibit higher mitochondrial biogenesis, but similar mitochondrial autophagy compared with fast-twitch muscles of *T. rubripes*, *n* = 3. (**A**) Mitochondrial DNA copy number of slow- and fast-twitch muscle. Expression of genes related to mitochondrial biogenesis (**B**) and autophagy (**E**) in slow- and fast-twitch muscle. Immunoblot (**C**) and densitometric quantification (**D**) of TFAM protein, which is normalized to GAPDH protein in slow- and fast-twitch muscles.

The mtDNA copy number is a marker of mitochondrial biogenesis, as mtDNA replication is a part of the mitochondrial biogenesis process. To reveal the mitochondrial biogenesis rate, we firstly analyzed the mtDNA copy number with mitochondrial gene NADH dehydrogenase 1(MT-nd1)—part of mitochondrial respiratory chain complex I—to enable NADH dehydrogenase (ubiquinone) activity, and nuclear gene *β-actin*. As shown in Figure 4A, slow-twitch muscle contains a higher mtDNA copy number compared with fast-twitch muscle. We then investigated the gene expression levels of two master regulators responsible for governing mitochondrial biogenesis: *Pgc-1α* (peroxisome proliferator-activated receptor-C coactivator-1α) and *Tfam* (transcription factor A, mitochondrial) [25]. As shown in Figure 4B, the mRNA expression levels of *Pgc-1α* and *Tfam* were higher in SM than FM. Furthermore, the protein levels of TFAM were also highly expressed in slow-twitch muscles (Figure 4C,D). Therefore, slow-twitch muscle exhibits higher mitochondrial biogenesis than fast-twitch muscle in *T. rubripes*, as indicated by a higher mtDNA copy number and elevated expression of mitochondrial biogenesis regulators. 

### 2.5. Mitochondrial Autophagy in SM and FM of T. rubripes Shows No Difference

We further analyzed the mRNA expression levels of mitochondrial autophagy-related *Pink1* (PTEN-induced putative kinase 1) and *Becn1* (Beclin1). To initiate mitochondrial autophagy, *Pink1* recruits autophagy receptors through phosphor–ubiquitin signaling [26]. Becn1 is a central player in the regulation of autophagosome formation and maturation [27]. As shown in Figure 4E, the lack of significant difference between *Pink1* and *Becn1* indicated that the autophagy level was not significantly different between slow- and fast-twitch muscles. 

### 2.6. Slow-Twitch Muscles Have Higher Mitochondrial Fusion and Fission Status Compared to Fast-Twitch Muscle

**Figure 5:**
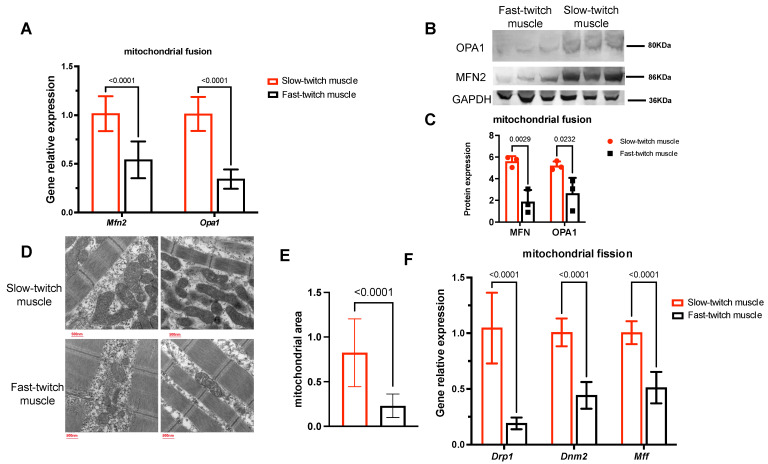
Slow-twitch muscles have higher mitochondrial fusion and fission rates compared to fast-twitch muscles, *n* = 3. (**A**) Expression of genes associated with mitochondrial fusion, including *mfn2* and *opa1*, between two types of muscle. (**B**,**C**) Protein expression of MFN2 and OPA1 were evaluated through Western blotting and normalized to GAPDH protein. The mean plus standard deviation of 3 biological replicates is shown. (**D**,**E**) Representative TEM images and quantification of mitochondrial area of two muscle types. (**F**) Expression of genes associated with mitochondrial fission, including *Drp1*, *Dnm2*, and *Mff*, in two types of muscle.

Besides biogenesis and autophagy, mitochondria also undergo a continuous process of fission and fusion to dynamically adjust their morphology according to the cellular environment [28]. Fusion leads to mitochondrial elongation and an increase in area, whereas fission fragments mitochondria and results in an increase in their number. We then investigated the role of mitochondrial fusion and fission in mitochondrial differences between SM and FM by examining the expression levels of key genes such as *Mfn2* and *Opa1* (involved in fusion) and *Drp1*, *Mff*, and *Dnm2* (involved in fission). As shown in Figure 5A–C, SM have significantly higher mRNA expression and protein levels of MFN2 and OPA1 compared to FM, indicating higher rates of mitochondrial fusion. This could result in mitochondrial elongation and then induce a larger mitochondrial area in SM compared with FM, as demonstrated in Figure 5D,E. Furthermore, the mRNA expression levels of *Drp1*, *Mff*, and *Dnm2* were also higher in slow-twitch muscles (Figure 5F), indicating higher mitochondrial fission which could further increase the mitochondrial number in the muscle. 

## 3. Discussion

**Figure 6:**
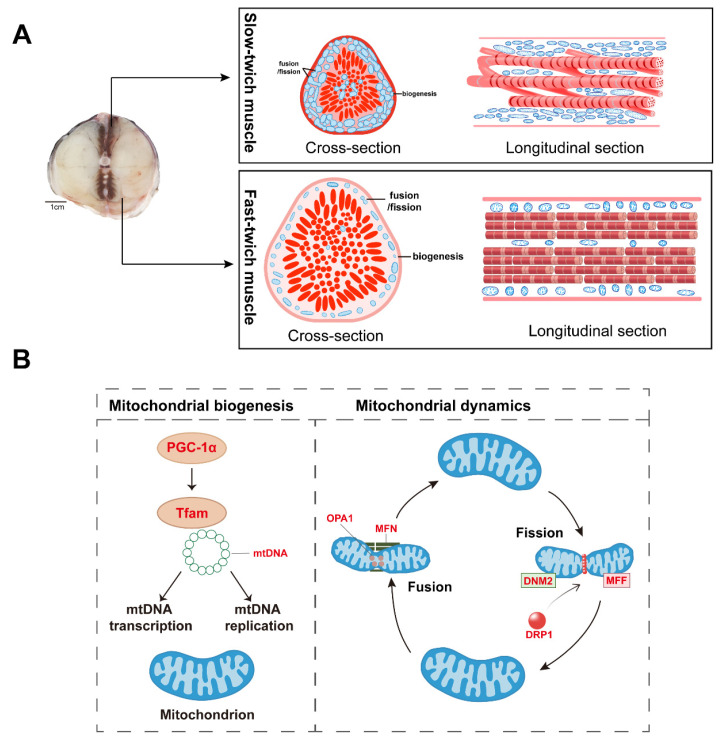
Mitochondrial distribution and its regulating mechanism in slow-twitch muscles and fast-twitch muscles of *Takifugu rubripes*. (**A**) Schematic diagram of muscle structure and mitochondrial distribution in slow-twitch muscles and fast-twitch muscles. The area of slow-twitch muscle fibers was smaller than fast-twitch muscle fibers. Fast-twitch muscle fibers were arranged more regularly than slow-twitch muscle fibers. The number and size of mitochondria in slow-twitch muscle fibers were greater than in fast-twitch muscle fibers, together with higher mitochondrial fusion, fission, and biogenesis rates. (**B**) Mitochondrial homeostasis process. Slow-twitch muscles express higher mitochondrial biogenesis and mitochondrial fusion and fission rates than fast-twitch muscles. Highly expressed genes in slow-twitch muscles are shown in red.

Skeletal muscle requires a constant energy supply for force production, with mitochondria strategically distributed within and around muscle fibers. The arrangement of mitochondria aids in distributing energy throughout the muscle reticulum, providing a rapid response mechanism to promptly adapt to fluctuations in energy demands [29,30]. The content, shape, and interconnectivity of mitochondria in different skeletal muscles adapt to the specific functional requirements of specialized fiber types. Teleost fishes are good models for studying skeletal muscles because their slow- and fast-twitch muscles are generally separated into anatomically distinct areas that can be easily distinguished by color. In this study, we used *T. rubripes* to investigate the number and morphology of mitochondria, as well as mitochondrial homeostasis, in SM and FM of teleosts. A schematic diagram of mitochondrial distribution and its regulating mechanism in slow- and fast-twitch muscles of *Takifugu rubripes* is shown in Figure 6. 

The continual ATP supply is essential in order to sustain skeletal muscle contraction. The order of muscle fiber recruitment is intricately influenced by the level of exercise intensity and contraction, thus determining the energy demand. During high-intensity efforts lasting seconds, the primary ATP source is derived from PCr breakdown and glycogen conversion to lactate (including glycogen breakdown and glycolysis). In contrast, activities lasting minutes to hours heavily rely on ATP generated from oxidative phosphorylation fueled by muscle glycogen and fatty acids [1]. In our studies, we found that slow-twitch muscles of *T. rubripes* contain larger amounts of mitochondria and rely more on slower, but more efficient aerobic metabolism, which meets the demands of high-power output with endurance and therefore predominates during sustained low-intensity swimming. Conversely, fast-twitch muscles contain fewer mitochondria and rely more on anaerobic metabolism for rapid ATP generation, making them better suited for short, explosive swimming [23,24]. Our results were consistent with previous TEM and immunofluorescence studies in humans, mice, and tilapias [4,15,31].

Maintaining a healthy mitochondrial population depends on mitochondrial homeostasis, which involves the coordination of finely-tuned mitochondrial turnover (biogenesis and mitophagy) with dynamic (fusion and fission) cycles [32]. To investigate the maintenance mechanisms of mitochondrial number and morphology in skeletal muscles of *T. rubripes*, we detected the homeostasis of the mitochondrial networks of SM and FM. Our data demonstrate that SM exhibited higher mitochondrial biogenesis and mitochondrial fusion/fission rates compared to fast-twitch muscles, and the markers of autophagy showed no difference between the two muscle types. 

Mitochondrial biogenesis is a process involving new mitochondrial material generation from pre-existing mitochondria, together with the synthesis of new mtDNA [6,7]. In skeletal muscles, mitochondrial biogenesis can expand the mitochondrial network and is critical for maintaining muscle performance and health [33]. Mitochondrial biogenesis is orchestrated by a multitude of transcriptional regulators within the cell. Notably, PGC-1α emerges as a central co-transcriptional regulator, driving mitochondrial biogenesis by interacting with Tfam [6]. In mice, PGC-1α was reported as a principle physiological regulator for slow-twitch muscles. Overexpression of PGC-1α in fast-twitch muscle stimulates its conversion to slow-twitch muscle, which is characterized by an upregulation of slow-twitch muscle-specific genes, greater resistance to electrically stimulated fatigue, and a distinct red color characteristic [34]. Tfam belongs to the high-mobility-group (HMG) protein family and is actively involved in mtDNA transcription, maintenance, and replication. Furthermore, the replication of mtDNA plays a significant role in driving mitochondrial biogenesis [35]. Consequently, the mtDNA copy number serves as an indicative marker of mitochondrial biogenesis [36]. And the interaction between TFAM and mtDNA holds significant relevance as it tightly regulates mitochondrial biogenesis [37]. In the present study, more abundant PGC-1α and Tfam, together with a larger mtDNA copy number, indicated higher a mitochondrial biogenesis rate in SM than FM. On the other hand, genes related to autophagy [38] showed no difference between the two muscle types, indicating that autophagy flux does not impact the mitochondrial number of the two types of muscles in *T. rubripes*. 

Besides mitochondrial biogenesis and autophagy, mitochondrial fusion and fission are crucially important mechanisms for mitochondrial function and quality control [39,40]. Fusion is the process wherein two mitochondria fuse their outer and inner membranes, resulting in the formation of a single and interconnected mitochondrion. On the contrary, fission allows segregation of mitochondrial components, thereby maintaining mitochondrial health [3]. Mitochondrial fusion machinery reduction in skeletal muscles causes severe mitochondrial dysfunction and mtDNA mutations which altogether drive muscle atrophy [41]. Muscles with defective mitochondrial fission decrease mitochondrial respiration, which thereby leads to severe muscle weakness and wasting, constituting the characteristic myopathic phenotype [28,42], whereas excessive expression of the mitochondrial fission machinery in adult muscle proves adequate to trigger muscle atrophy [43,44]. The studies mentioned above indicate the critical role of mitochondrial fusion and fission in skeletal muscle, as they are essential for sustaining a highly interconnected functional mitochondrial network and thus maintaining healthy skeletal muscle function. 

In the present study, slow-twitch muscles showed higher expression levels of the genes involved in mitochondrial fusion and fission than fast-twitch muscles in *T. rubripes*. Furthermore, the mitochondria are elongated and interconnected in slow-twitch muscles, whereas they are punctate and isolated in fast-twitch muscles. Several studies found that the mitochondrial fusion and fission balance plays a critical role in shaping mitochondrial morphology, which in turn is important for maintaining mitochondrial function. This balance emerges as a significant factor influencing muscle mass maintenance, homeostasis, and metabolic processes [4,42]. The elongated, interconnected mitochondrial architecture of slow-twitch muscles facilitates the transfer of high-energy ATP from the mitochondria to the contractile apparatus [25]. In *T. rubripes*, we suspect that dynamic regulation of fission–fusion events adapts mitochondrial morphology to the specific bioenergetic requirements of different fiber types. Elongated mitochondrial networks, together with higher mitochondrial fusion and fission rates, are associated with increased oxidative metabolic states [45,46,47] and support the maintenance of ATP levels in SM, satisfying prolonged energy production in teleosts.

In summary, we proved that distinctions in mitochondrial homeostasis rates, together with mitochondrial number and morphology, are defining characteristic between SM and FM of teleost fishes. Slow-twitch muscles contain larger numbers and more elongation of mitochondria according to the TEM results, together with high mitochondrial-specific protein expression and high mitochondrial-encoded gene expression. To maintain the number and morphology of mitochondria, slow-twitch muscles perform higher rates of mitochondrial biogenesis and mitochondrial fusion/fission. Therefore, we suspect that the larger numbers and greater elongation of mitochondria in slow-twitch muscles adapt to the bioenergetic requirements of slow-twitch muscles that require long-term energy production in *T. rubripes*. 

A series survey among various teleost fishes revealed a positive correlation between the proportion of slow-twitch muscle and sustained swimming activity [48,49]. Species characterized by greater activity levels exhibit a relatively larger proportion of slow-twitch muscle compared to their sedentary counterparts. Higher proportions of slow-twitch muscle containing more mitochondria could provide power with endurance and efficiency for sustained swimming. As mitochondrial structure and number could correspond to movement level in mammals, we suspect that mitochondrial homeostasis adapts muscles to the bioenergetic requirements of teleost fishes with various swimming performances. In future research, it will be important to address the role of mitochondrial homeostasis in the adaptation of skeletal muscle to the various swimming performances of teleost fishes.

## 4. Materials and Methods

### 4.1. Fish Samples and Tissue Collection

We procured 3 adult *Takifugu rubripes* specimens from Dalian Tianzheng Industrial Co., Ltd. (Dalian, Liaoning, China). Following anesthesia using 20 mg/L MS-222 (tricaine methane sulfonate), the body weights and lengths of the fishes were determined (Table S1). SM and FM samples were promptly collected for subsequent experiments. Immediately after sampling, the fishes were euthanized with overdose anesthetics. 

### 4.2. Histological Analysis 

Slow- and fast-twitch muscle samples were fixed in 4% PFA (paraformaldehyde) solution for 24 h, followed by a progression through an ethanol series for dehydration, xylene treatment for dewaxing, and paraffin embedding. The samples were then sliced transversely into 4-μm-thick sections to facilitate H&E and HSP60 IHC staining, following standard procedures. Image acquisition utilized a Leica microscope (Leica, Heidelberg, Germany), while subsequent analysis was conducted via ImageJ2 (NIH, Bethesda, MD, USA).

### 4.3. Hsp60 Immunofluorescence Staining

HSP60 is a mitochondrial chaperone protein that can serve as a marker for mitochondrial number. We conducted HSP60 immunofluorescence staining on these muscle types. HSP60 primary antibody (GB11243, Servicebio, Wuhan, China) was incubated overnight at 4 °C for IHC staining, and the goat anti-rabbit Alexa 594 secondary antibody was applied for visualization. Nuclei were stained with DAPI. 

### 4.4. Transmission Electron Microscopy 

SM and FM samples were fixed in a 2.5% glutaraldehyde solution for 12 h. After carefully being washed with phosphate-buffered saline (PBS) solution, samples underwent post-fixation using a 1% osmium tetroxide solution for 2 h followed by a careful 15 min wash. The fixed samples were then dehydrated with increasing concentrations of acetone. Then, samples were embedded within epoxy resin (SPI Chem/SPI-PON 812 KIT, West Chester, PA, USA). Next, semi-thin sections (1 μm) of embedded tissue were treated with 1% toluidine blue to assess localization. Ultrathin sections were then sliced at 70 nm and collected in 150-mesh grids. Then, 3% uranyl acetate–lead citrate was used to double stain the grids. The ultrastructure of mitochondrial morphology was photographed using a JEM-1200EX transmission electron microscope (JEOL, Tokyo, Japan).

### 4.5. Western Blot Analysis 

Skeletal muscle tissues were lysed in RIPA buffer containing PMSF. The quantification of proteins was executed using a BCA (bicinchoninic acid) protein assay kit (PA115, TIANGEN, Beijing, China). Proteins were separated via SDS-PAGE using 4–12% precast gels (M00653, Genscript, Nanjing, China) followed by blotting onto nitrocellulose membranes. To block nonspecific binding, membranes were immersed in 5% non-fat milk solution in TBST (Tris-buffered saline containing 0.1% Tween-20) for 1 h. Primary antibodies used in the present study are shown in Table 1. Then, HRP-conjugated secondary antibodies (goat anti-rabbit or goat anti-mouse) were applied for 1h. Protein bands were visualized with the DAB chromogenic reagent kit (PA110, TIANGEN) and protein expression levels were analyzed using ImageJ. 

### 4.6. mRNA Expression Analysis

Using q-RT-PCR, we measured the mRNA levels of genes encoded by mitochondrial genomes and genes involved in the mitochondrial homeostasis process. The primers used in the present study are listed in Table 2. Total RNA was extracted from both SM and FM samples and subsequently reverse transcribed into cDNA. Gene expression levels were assessed using RT-PCR (real-time quantitative reverse transcription PCR) through the 2^−ΔΔCt^ method [19].

### 4.7. Mitochondrial DNA Copy Number Analysis

Total DNA extraction from slow- and fast-twitch muscles was achieved using the DNA extraction kit (TIANGEN, Beijing, China), according to the manufacturer’s guidelines. The mitochondrial DNA (mtDNA) copy number was tested through quantification of the mitochondrial marker gene NADH dehydrogenase 1 (*nd1*), which encodes a core subunit of the mitochondrial respiratory complex I. The mtDNA copy number was determined through RT-PCR and normalized against a nuclear-encoded *β-actin* gene, as previously described [20]. Specific primer sequences can be found in Table 2.

### 4.8. Gene Expression Heatmap of Energy Metabolism-Associated Genes

Mitochondria are sites of aerobic metabolism, while anaerobic metabolism occurs in the cytoplasm of cells, so the aerobic metabolism level can reflect the mitochondrial number within tissues. To further investigate the mitochondrial differences between SM and FM, we analyzed the expression of genes related to anaerobic and aerobic metabolism in these two muscle types using transcriptome data. The transcriptomic data of SM and FM in adult *T. rubripes* were downloaded from NCBI with the accession number SRX2413542 and SRX2413433 [17]. Then we used the pheatmap package to explore the differential high- and low-expression genes involved in energy metabolism, including anaerobic and aerobic metabolism, in slow- and fast-twitch muscles.

## Figures and Tables

**Table 1:** Antibodies used in this study.

Protein Name	Cat. Num	Company
Hsp60	GB11243	Servicebio, Wuhan, China
Tfam	22586-1-AP	Proteinintech, Tokyo, Japan
GAPDH	GB11243	Servicebio, Wuhan, China
OPA1	612606	BD, Franklin Lakes, NJ, USA
MFN2	ab56889	Abcam, Cambridge, UK

**Table 2:** Primers used in this study.

Gene	Sequences (5′-3′)	Application
*nd1*	TACTAGCCGTAGCTTTCTTA	mtDNA copy number analysis
	GGTCGGACTGGTTCTTT	
*β-actin*	CCAGAAAGACAGCTACGTTGG	
	GCAACTCTCAGCTCGTTGTAG	
*coi*	TGATTGGAGGCTTTGGGA	mitochondrial-encoded genes
	CAGAAGGAGGAAGGATGG	
*cytb*	ACGATTCTTTGCCTTCCA	
	AATAGGGCGAGTGTTGC	
*fis1*	GCAAATACACGGAGGACAT	mitochondrial fission
	CTTCAGGGCTTTCTCGTAG	
*drp1*	CATTTCAAACCCAAACTCCA	
	CGCATCTGCCACCAAC	
*mfn2*	GCCACTCAAGCATTTCGTC	mitochondrial fusion
	CTCGTCATTCTTGTGAGTATCT	
*opa1*	CCGCACACAGTTAAAATATCAG	
	TGGTCCTGGGTGTTGTAG	
*atg5*	CGAGCCTTACTATCTGTTGC	mitochondrial autophagy
	GTCCCTTCATACTCAAACCA	
*pink1*	CTGGTGGTCAGTGACTTTGGC	
	GCGTCCGCTTTGCTGTAG	
*pgc-1α*	GGCGACATTGCACCGAGTT	mitochondrial biogenesis
	GCCCTTGGCGTCGTTATTT	
*tfam*	AAGCGTCCTCGCTCAC	
	TTAGGTCTTCCCGTCCA	
*rpl19*	GTCTCATCATCCGCAAACC	reference gene
	TCTCAGGCATACGAGCATT	

## Data Availability

Data are contained within the article and Supplementary Materials.

## Supplementary Materials

The supporting information can be downloaded at: https://www.mdpi.com/article/10.3390/ijms25031512/s1.
