# Supplementary material for: Mitochondrial Homeostasis Regulating Mitochondrial Number and Morphology Is a Distinguishing Feature of Skeletal Muscle Fiber Types in Marine Teleosts
Source: Int J Mol Sci. 2024 Jan 26;25(3):1512. doi: 10.3390/ijms25031512 (PMC10855733; doi:10.3390/ijms25031512)
Supplement: Supplementary file 1 [file ijms-25-01512-s001.zip › ijms-2806418-supplementary-done.pdf]

International Journal of *Molecular Sciences*

MDPI

Article

Mitochondrial Homeostasis Regulating Mitochondrial Number and Morphology Is a Distinguishing Feature of Skeletal Muscle Fiber Types in Marine Teleosts

Busu Li [1,2], Huan Wang [1], Xianghui Zeng [1], Shufang Liu [1,2,*] and Zhimeng Zhuang [1]

[1] National Key Laboratory of Mariculture Biobreeding and Sustainable Goods, Yellow Sea Fisheries Research Institute, Chinese Academy of Fishery Sciences, Qingdao 266071, China
[2] Laboratory for Marine Fisheries Science and Food Production Processes, Laoshan Laboratory, Qingdao 266237, China
* Correspondence: liusf@ysfri.ac.cn

Supplementary Materials

Table S1. The body weight and length of *Takifugu rubripes* used in present study.

Species	Sample Number	Body Weight (g)	Full Length (cm)
	1	1317.8	37.5
Takifugu rubripes	2	1059	33.5
	3	2241	44
